# Investigating Vernal Pool Fairy Shrimp Exposure to Organophosphate Pesticides: Implications for Population-Level Risk Assessment

**DOI:** 10.3390/ecologies3030024

**Published:** 2022-08-02

**Authors:** David H. Miller, Matthew Etterson, Leah Oliver, Elizabeth Paulukonis, Nathan Pollesch, S. Thomas Purucker, D. Christopher Rogers, Sumathy Sinnathamby, Sandy Raimondo

**Affiliations:** 1 United States Environmental Protection Agency, Office of Research and Development, Great Lakes Toxicology and Ecology Division, Duluth, MN 55804, USA; 2 United States Environmental Protection Agency, Office of Research and Development, Gulf Ecosystem Measuring and Monitoring Division, Gulf Breeze, FI 32561, USA; 3 Kansas Biological Survey and the Biodiversity Institute, The University of Kansas, Lawrence, KS 66047, USA; 4 United States Environmental Protection Agency, Office of Chemical Safety and Pollution Prevention, Office of Pesticide Programs, Washington, DC 20004, USA

**Keywords:** *Branchinecta lynchi*, Pop-GUIDE, population model, chemical stress, climate change

## Abstract

Vernal pool fairy shrimp, *Branchinecta lynchi*, is a freshwater crustacean endemic to California and Oregon, including California’s Central Valley. *B. lynchi* is listed as a Federally Threatened species under the US Endangered Species Act, and as a vulnerable species on the IUCN Red List. Threats that may negatively impact vernal pool fairy shrimp populations include pesticide applications to agricultural land use (e.g., agrochemicals such as organophosphate pesticides) and climate changes that impact vernal pool hydrology. Pop-GUIDE (Population model Guidance, Use, Interpretation, and Development for Ecological risk assessment) is a comprehensive tool that facilitates development and implementation of population models for ecological risk assessment and can be used to document the model derivation process. We employed Pop-GUIDE to document and facilitate the development of a population model for investigating impacts of organophosphate pesticides on vernal pool fairy shrimp populations in California’s Central Valley. The resulting model could be applied in combination with field assessment and laboratory-based chemical analysis to link effects from pesticide exposure to adverse outcomes in populations across their range. *B. lynchi* has a unique intra-annual life cycle that is largely dependent upon environmental conditions. Future deployment of this population model should include complex scenarios consisting of multiple stressors, whereby the model is used to examine scenarios that combine chemical stress resulting from exposure to pesticides and climate changes.

## 1. Introduction

During the wet phase (November–March), vernal pools in California are habitat for up to six crustaceans that are federally listed endangered and threatened species (listed species) such as the vernal pool fairy shrimp (*B. lynchi*) and the vernal pool tadpole shrimp (*Lepidurus packardi*) [1–3]. Inundated vernal pools are utilized by many species of birds, amphibians, reptiles, and mammals as sources of water, food, and as breeding sites, including 11 listed species across these groups. During the flowering phase (March–May), vernal pools provide critical habitat for 39 listed plant species, such as Boggs lake hedge-hyssop (*Gratiola heterosepala*) and Solano grass (*Tuctoria mucronata*). In addition to providing habitat to listed species, vernal pools offer a number of ecosystem services, such as water regulation/flood water storage, water purification, and aesthetic/cultural value [4].

California vernal pools have been highly impacted by human activities, including conversion to urban, commercial, industrial and agricultural use [5,6]. Pesticide use is documented to potentially impact vernal pool biodiversity in California [7,8]. Many vernal pools are in or near agricultural areas, and may be exposed to pesticides via runoff, drift, and direct spray. Pesticides can enter vernal pool systems as wet deposition via rainfall, or as surface water loads from adjacent agricultural lands [8–12]. Using a weight of evidence community-level approach, Raimondo et al. (2019) demonstrated that for insecticides, targeting protections to the vernal pool fairy shrimp and its habitat would also afford protections from future effects to the community of listed species and ecosystem services associated with this habitat [4].

Climate changes including increased temperature and CO_2_ are likely to expand pest ranges and extending crop growing seasons, potentially resulting in increased pesticide applications which could lead to risks to vernal pool organisms ([13]. Complex relationships between climate change and pesticide impacts are likely, including interactions between climate stressors and toxicological responses of exposed organisms [14]. Climate change will impact environmental concentrations of pesticides due to a combination of increased volatilization and accelerated degradation, both which are affected by moisture content, elevated temperatures and direct exposure to sunlight [14].

Organophosphate insecticides are commonly used in California’s Central Valley, and have been detected in air, snow, and surface waters [15–18]. Two examples are malathion and diazinon, which are used on a variety of applications including orchards, terrestrial food and feed crops, terrestrial nonfood crops, as well as in greenhouse growing operations, and non-agricultural locations. Diazinon, and malathion application in California’s Central Valley may have the potential to adversely impact *B. lynchi* in vernal pools based upon USEPA Office of Pesticide Programs (OPP) aquatic life benchmark values, California Regional Waterboard Aquatic Life Criteria, and predicted values for the vernal pool fairy shrimp from surrogate species toxicity values using the Web-based Interspecies Correlation Estimation application (Web-ICE) [4,19].

Population models provide a mathematical method to integrate multiple sources of stressors. For example, toxicological effects that are measured at the organismal level can result in changes to vital rates (i.e., survival and reproduction) that can be incorporated with effects of other stressors to evaluate impacts to populations or subpopulations at larger scales. The application of population models is useful for both ecological risk assessment and population management (e.g., [20–25].

Guidance for population model development and application have addressed many of the challenges associated with their implementation and aim to facilitate the integration of population modeling into standard ecological risk assessment (ERA) practices [22,26–28]. Pop-GUIDE (Population model Guidance, Use, Interpretation, and Development for Ecological risk assessment) is one such tool that uses the objective of the risk assessment to guide model development and serves as documentation of the model development process. The conceptual model developed within Pop-GUIDE facilitates model implementation and evaluation, allowing for consideration of model requirements, objectives, and complexities of protection goals [28]. It also allows for transparent model development by explicitly presenting key decisions and assumptions made during development. Trade-offs associated with generality, realism and precision are considered with respect to resources, data availability, and risk assessment objectives to ensure the resulting uncertainties of the final model are consistent with those of the ERA [28]. In the present study, we employ Pop-GUIDE to develop a population model that can be used to evaluate the potential effects of exposure to organophosphate pesticides to the listed species *B. lynchi* in vernal pools located in the Central Valley of California.

Model credibility is critical for the implementation of a model into an ecological risk assessment. The present study provides a suitable method to transparently convey model development to stakeholders, and is a demonstration of how Pop-GUIDE could be employed for assessing ecological risk. Our population model could be utilized in conjunction with ongoing field monitoring efforts (e.g., through effects-based monitoring programs) and/or laboratory analysis to link effects resulting from pesticide exposure to adverse outcomes in vernal pool fairy shrimp populations across their range. Furthermore, this study comprises an important advancement in the consideration of population model development for a species with an intra-annual life cycle that is highly dependent upon environmental conditions and a dormant, long term egg bank. Future deployment could include complex scenarios consisting of multiple stressors to examine a population exposed to both chemical and/or non-chemical stressors (i.e., climate change).

## 2. Methods

### 2.1. Overview of Life History Relevant to Model Development

Vernal pools are shallow depressions of varying size which are underlain by an impervious substrate that ensures a perched water table. Water fills vernal pools in winter and then evaporates by late spring, leaving a cracked mud flat [29]. California’s vernal pools may vary enormously in size, ranging from a few square meters to more than 100 hectares in area and with depths that can exceed one half meter [29]. Vernal pool fairy shrimp are specifically adapted for fluctuating wet and dry conditions, which are determined by the season and amount of rainfall [3,30,31]. Vernal pool wet season is from November to March during which time the fairy shrimp are in mobile stages. When pools are dry from March through October, fairy shrimp are dormant within the egg bank. The life cycle can be represented as three stages: eggs, immature, and adult. All *B. lynchi* populations are each limited to their individual vernal pool that is hydrologically isolated preventing direct active dispersal. Maturity is reached as early as 12d, and the adult stage can last for multiple weeks. Adults produce eggs that require desiccation before they can hatch, and the diapausing eggs lie dormant in or on the substrate during the dry season, hatching when liquid water is present ([30,32]. Eggs may hatch the year after they are laid, or they may remain dormant for up to several years as a bet hedging strategy. While the immature and adult stages typically do not move from one vernal pool to another, studies have demonstrated that vernal pool fairy shrimp can be dispersed from their habitats passively as eggs by pagile vectors such as mammals, birds, fish, amphibians, crayfish, insects and wind [31,32]. Immigration and emigration of *B. lynchi* eggs between vernal pools may function to preserve habitats with greater exposure to stressors. Rogers (2015a) found that the spatial distribution of Anostraca habitats (basins) drive island biogeographical patterns depending upon the relative isolation and size of any one given pool [32].

### 2.2. Utilization of Pop-GUIDE for Model Development

Pop-GUIDE is novel as it uniquely brings together the decision guide developed by Schmolke et al. (2017) and the modeling framework proposed by Raimondo et al. (2018) in conjunction with the recommendations of European Food Safety Authority (EFSA) as a comprehensive tool for implementation [22,26,27]. Pop-GUIDE provides a stepwise process to assist development of a model that is appropriate for the assessment objective and available data. A series of five sequential phases were utilized following the guidance from Pop-GUIDE that included: Model Objectives (Phase 1), Data Collection (Phase 2), Decision Steps (Phase 3), Conceptual Model (Phase 4), and Evaluation and Implementation (Phase 5) [28].

#### 2.2.1. Phase 1: Model Objectives

For the present study, we developed a model construct capable of employment for the pursuit of an established an ERA objective as follows: to determine if exposure to organophosphate pesticides may decrease populations of *B. lynchi*. From this, we identified a model objective that included an exploration of the trade-offs of generality, realism, and precision associated with an ERA objective for vernal pool fairy shrimp [27,28,33]. Generality is desirable for ERA in which the objectives do not target specific locations, habitats, or species. The specificity of these attributes increases with increasing realism. Precision corresponds to the level of confidence in the data available to inform model processes [28]. We employed the decision tree from Pop-GUIDE to first identify the trade-offs of generality, realism, and precision of the ERA based on the inclusion or exclusion of listed species as one function of taxonomic specificity, and as well as the geographic scale of our assessment. In using the decision tree, the trade-off category was identified (Pop-GUIDE allows for five trade-off categories including general, realistic, general-realistic, general-precise, realistic-precise). A standard series of questions provided in Pop-GUIDE Phase 1 were used to translate the ERA objective and trade-offs into those for model building [28]. These questions are listed in Table 1 and were used in translating ERA trade-offs to those of the model to be used in its context.

#### 2.2.2. Phase 2: Data Collection

A comprehensive description of the life history and habitat of the focal species, as well as exposure and effects information, is critical to a population modeling study for which ecological risk is evaluated. We followed Pop-GUIDE methods to organize available pertinent data via tables that group characteristics as Organism-Level Characteristics, Population-Level and Spatial Characteristics, External Factors and Habitat Characteristics, and Exposure and Effects Characteristics [28]. Data were collected and evaluated for each given characteristic and then classified as general, realistic, and precise, reflecting data specificity, variability and/or confidence. Data taxonomic specificity and corresponding references were documented. Data collection included studies from both laboratory and field.

#### 2.2.3. Phase 3: Decision Steps

To identify which data, functions, and relationships to include in the model, we employed a series of decision steps using the Phase 3 of Pop-GUIDE [28]. These decision steps are organized across five broad topics that included (I) Life history representation, (II) Organism-level processes, (III) Population and spatial factors, (IV) External factors and (V) Exposure and effects. Developing a life history representation was an important step in the vernal pool fairy shrimp model development, as it provided a visual summary of the critical life stages and demographic processes driving the species’ dynamics. Decisions regarding organism-level processes were made in relation to growth, development, maturation and reproduction in the model. The investigation of population and spatial factors focused on analysis of population status, density dependence, movement, and habitat characteristics. Evaluation of external factors included interspecific interactions, abiotic factors, and environmental conditions to model development, as well as available exposure and effects data (i.e., acute and chronic toxicity values and endpoints affected) and temporal resolution of the model.

#### 2.2.4. Phase 4: Conceptual Model

Proceeding forward using life history *of B. lynchi* and the analyses of Phases 2 and 3, we identified the components to include in a conceptual model. The conceptual model provided a graphical and textual summary of the compartments, linkages, and/or functions within the vernal pool fairy shrimp population model. Information specific to vernal pool fairy shrimp exposed to pesticides in California’s Central Valley were included where available, and in combination with data for surrogate species (i.e., *Thamnocephalus platyurus*) that were used to inform some processes and components when data for *B. lynchi* were not available.

#### 2.2.5. Phase 5: Evaluation and Implementation

Within Pop-GUIDE, model implementation and evaluation in Phase 5 is pursuant to the extension of the conceptual model to a fully parameterized computational tool, including model analysis for evaluation of behavior and performance (i.e., uncertainty analysis, sensitivity analysis, and how it could be used for regulatory purposes) [28]. Within the scope of the present study, we built upon the data evaluation conducted in Phase 2 and the theoretical concepts identified in Phase 3 and described how the conceptual model of Phase 4 could be implemented within the context of a risk assessment for *B. lynchi*. Uncertainty analysis can be used to describe the influence of model variables or processes on the model outputs, such as those linked to model structure, and/or parameter estimation as a result of measurement errors, biological variability, or extrapolation among species or environments [26]. We explored uncertainty that would be foreseeable based upon completing Phases 1 through 4 of Pop-GUIDE, and we identified independent empirical data that would be needed to validate model predictions. Mathematical formalism was not considered and thus a quantitative model implementation and evaluation (including a sensitivity analysis and a more detailed uncertainty analysis) is a subsequent next step beyond the scope of the present study.

## 3. Results

### 3.1. Utilization of Pop-GUIDE for Model Development

#### 3.1.1. Phase 1: Model Objectives

**Figure 1.**
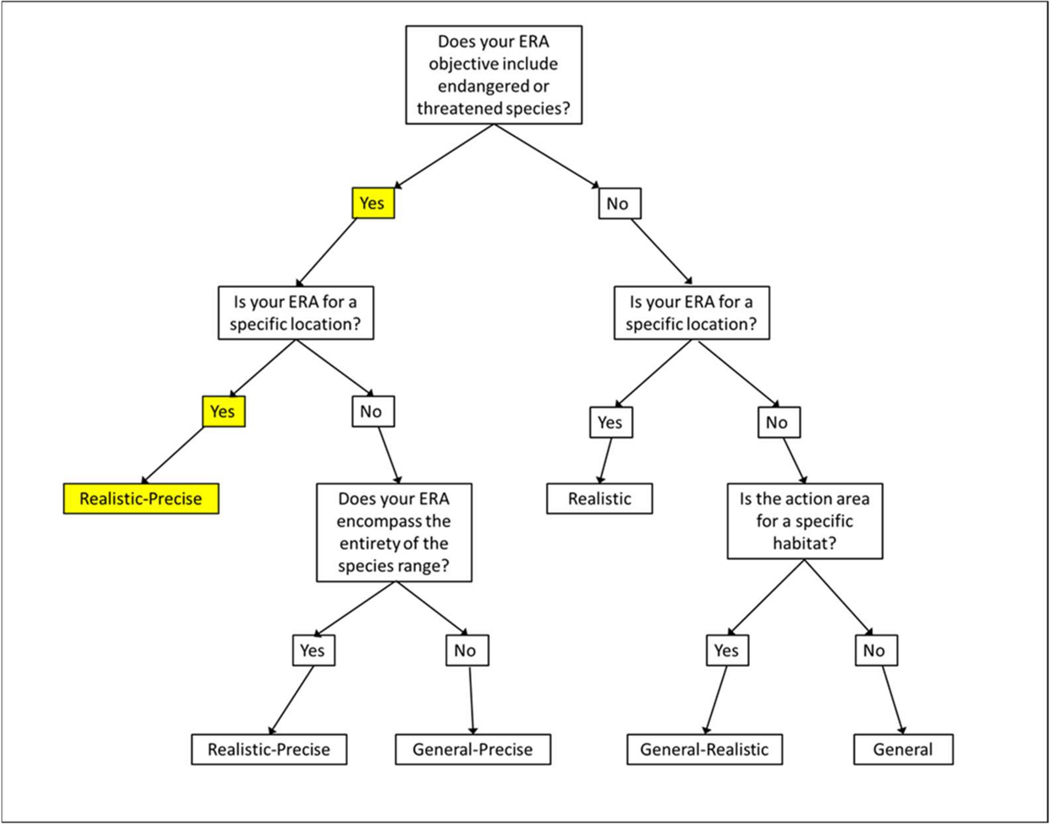
Decision tree taken from Pop-GUIDE. Decisions made for each question are highlighted in yellow.

The decision tree taken from Pop-GUIDE (Figure 1) identified the category of trade-offs for the ERA objective. Since the ERA is to evaluate the risk to the vernal pool fairy shrimp, a threatened species, and the ERA is for a specific location, i.e., California’s Central Valley, the trade-off category of the assessment is Realistic-Precise. To translate this ERA category into model objectives, we applied the standard questions taken from Pop-GUIDE (Table 1). In response to Question 1, ideally the population model will be used in an ERA as a direct assessment tool and model outputs should be precise and realistic. The ERA should include relevant temperature and rainfall profiles, as well as exposure data to the specific organophosphate pesticide(s) of interest at the location. In answering Question 2, annual population abundance and production of eggs will be assessment endpoints that are most relevant to the ERA objective and the intended model use. Endpoints of secondary importance would include population growth rate and probability of population decline. Model parameterization of vital rates that interact with important environmental variables should be as precise as possible. In considering Question 3, it is important to note that vernal pools are influenced by astatic and episodic weather patterns (i.e., temperature and rainfall). Application of organophosphate pesticides may also be temporal (seasonal), so it will be important to determine if pesticide application temporally overlaps with pond inundation. In responding to Question 4, as the ERA category is realistic-precise, the model is applicable to the entire Central Valley and is capable of being applied in a metapopulation context. In this context, realism is favored while sacrificing precision. However, the spatial resolution can be narrowed thereby yielding more precise predictions for a specific pool or refined area. Uncertainty associated with estimation of vital rates (i.e., reproduction and survival), egg dispersal rate (i.e., wind, wildlife, predators), pesticide application (timing, amount, transport route), and weather patterns (e.g., rainfall timing and amount) should be considered. As the focus of this effort is on a listed species, which requires a high level of certainty in risk estimation, model results should be represented by quantitative confidence bounds derived from empirical functions within the model. In answering Question 5, the model is being constructed with existing data collected from both the field and laboratory. There is no time or budget established to collect additional data. Overall, based on the answers to these five questions, the model objective was determined to be to provide reliable estimates of potential impacts of realistic pesticide exposure scenarios on vernal pool fairy shrimp abundance using available data.

#### 3.1.2. Phase 2: Data Collection

Data were assembled for organism-level characteristics including life span, reproductive measures (breeding season, frequency, and clutch size), onset of maturation, hatching rate, immature transition rate, sex ratio, recruitment rate, survival rate, growth rate, and egg emergence [5,31,32,34–42] (see Table S1). Some characteristics such as survival and onset of maturation were temperature dependent [34,35,42]. Further, the reproductive season of vernal pool fairy shrimp was observed to be correlated with pool inundation depth and duration [34,36–38,41]. Thus, weather patterns including spatial and temporal trends in temperature and precipitation were important in population model development (i.e., in estimation of vital rates). Data collected for population and spatial characteristics included density dependence, population size, metapopulation structure, movement, geographic range and habitat measures (features and classification/suitability) [1,5,8,24,31,32,34,35,43–47] (see Table S2). Density dependence (e.g., based upon pond volume) was important to consider due to potential impact on fitness [35]. Mobile stages do not move from one vernal pool to another; however the eggs are capable of being dispersed passively from their habitats by pagile vectors such as mammals, birds, fish, amphibians, crayfish, insects and wind [31,32].

Data amassed for external factors included interspecific interactions (predation and competition), environmental conditions, stressors, and existing management of the species [1,5,31,32,34,48,49] (see Table S3). While vernal pool fairy shrimp can cooccur with other shrimp species, competition is unlikely to be important [5]. The importance of environmental conditions (e.g., temperature and duration of pool inundation) is well documented and including explicit relationships between these variable and fairy shrimp dynamics may assist in understanding how climate change may impact population sustainability. For example, climate changes could exacerbate impacts due to chemical exposure if the vernal pools do not get charged as frequently and/or may not stay inundated for as long [50]. Data acquired for chemical exposure includes estimated environmental concentrations from the Pesticide Water Calculator (PWC version 1.59), which has been used to predict temporal trends of organophosphate pesticide concentrations (including diazinon and malathion) in three CA vernal pools based upon exposure duration and representative of nearby crop application [8]. Chemical effects characteristics included representation of toxic effects using standard crustacean surrogates (e.g., *Daphnia magna, Thamnocephalus platyurus*) and examination of effects by life stage or size as well as exposure route [4,7,8,15–19,51–57] (see Table S4).

#### 3.1.3. Phase 3: Decision Steps

**Figure 2.**
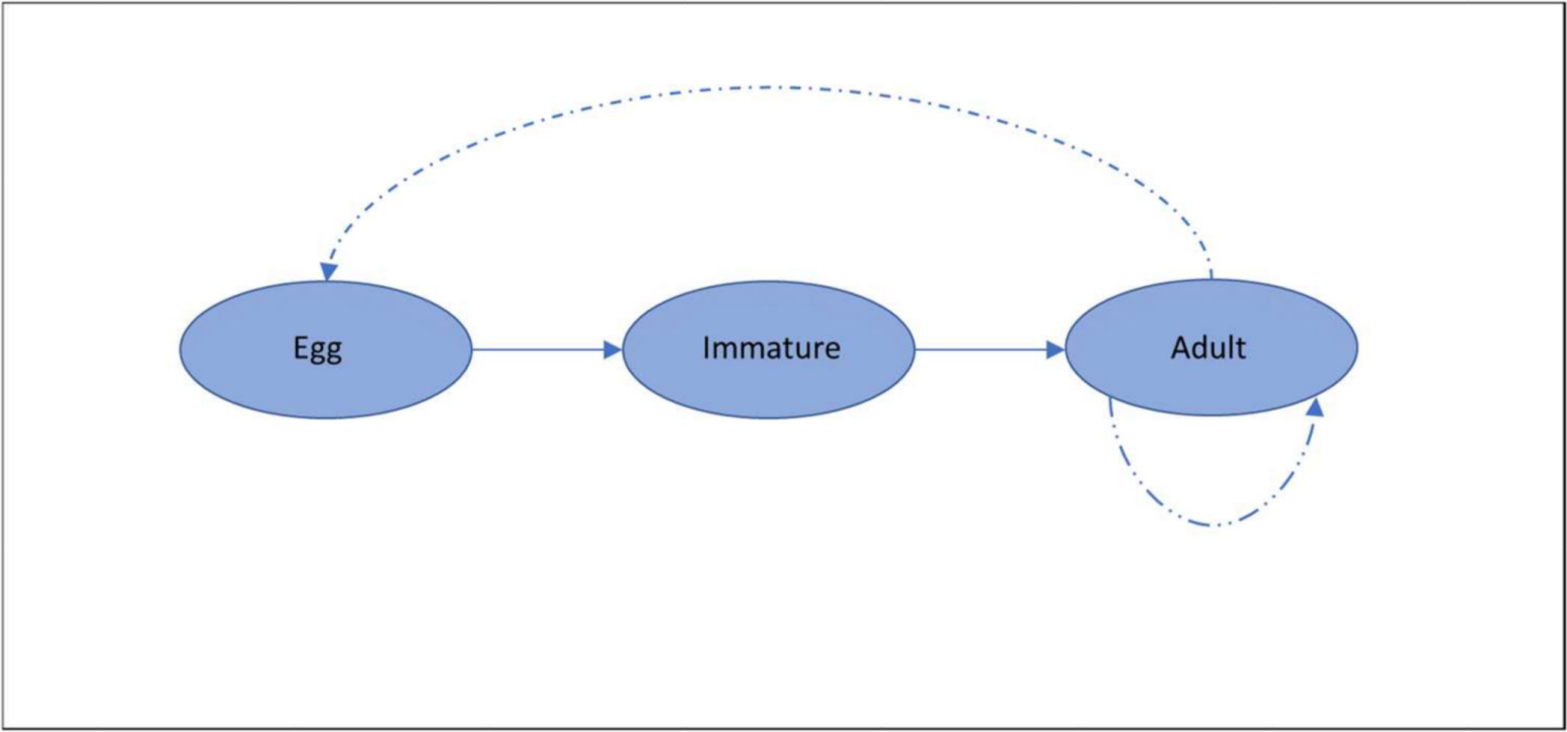
The life history of vernal pool fairy shrimp.

The life history of vernal pool fairy shrimp is represented by Figure 2, whereby egg, immature and adult stages comprise the life cycle. We used the keys published within Pop-GUIDE for analysis (decisions we made are highlighted in yellow) of the remaining decision steps [28]. Using this key, we worked through a series of preset questions and recorded our decisions made for each question (decisions we made are highlighted in yellow) for each remaining step (see Figure S1). For step II, Organism-level processes (see Figure S1, Panel A), it was determined that there was not sufficient information to represent growth continuously, and that both maturation and fecundity could be modeled as dependent upon age. In examining step III, Population and spatial factors (see Figure S1, Panel B), it was determined that environmental stochasticity and variation in demographic rates should be included in model applications. Further, it was decided that a consistent density dependent function should be implemented across all life stages, such as the logistic function implemented across all age classes used in modeling other crustacean species [24]. Following the decision steps, migration/dispersal of adults, behavior, and consideration of multiple habitat types did not need to be represented in the model. In evaluation of step IV, External factors (see Figure S1, Panel C), environmental conditions were determined to be important drivers of the population dynamics and dependence on environmental condition should be represented in the model. Variation in across-year environmental conditions may be captured by environmental stochasticity in the model that is derived from the variation of measured temperature and rainfall from past years. Based on the life history of the vernal pool fairy shrimp, neither diet nor other obligatory relationships needed to be included in model conceptualization and formulation. In assessment of step V) Exposure and effects characteristics (see Figure S1, Panel D), it was determined that exposure should be included via direct contact in aqueous solution as measured in tests. Effects on survival should be represented in the model using a threshold (i.e., based upon NOEC/LOEC estimates), and sublethal effects should not be represented in the model at this time based upon available data (effects on fecundity could be included if warranted by future laboratory analysis). It was also decided that seasonal differences (i.e., seasonal patterns associated with the use of pesticides on agricultural land) would be important to adequately describe species and chemical co-occurrence.

#### 3.1.4. Phase 4: Conceptual Model

**Figure 3.**
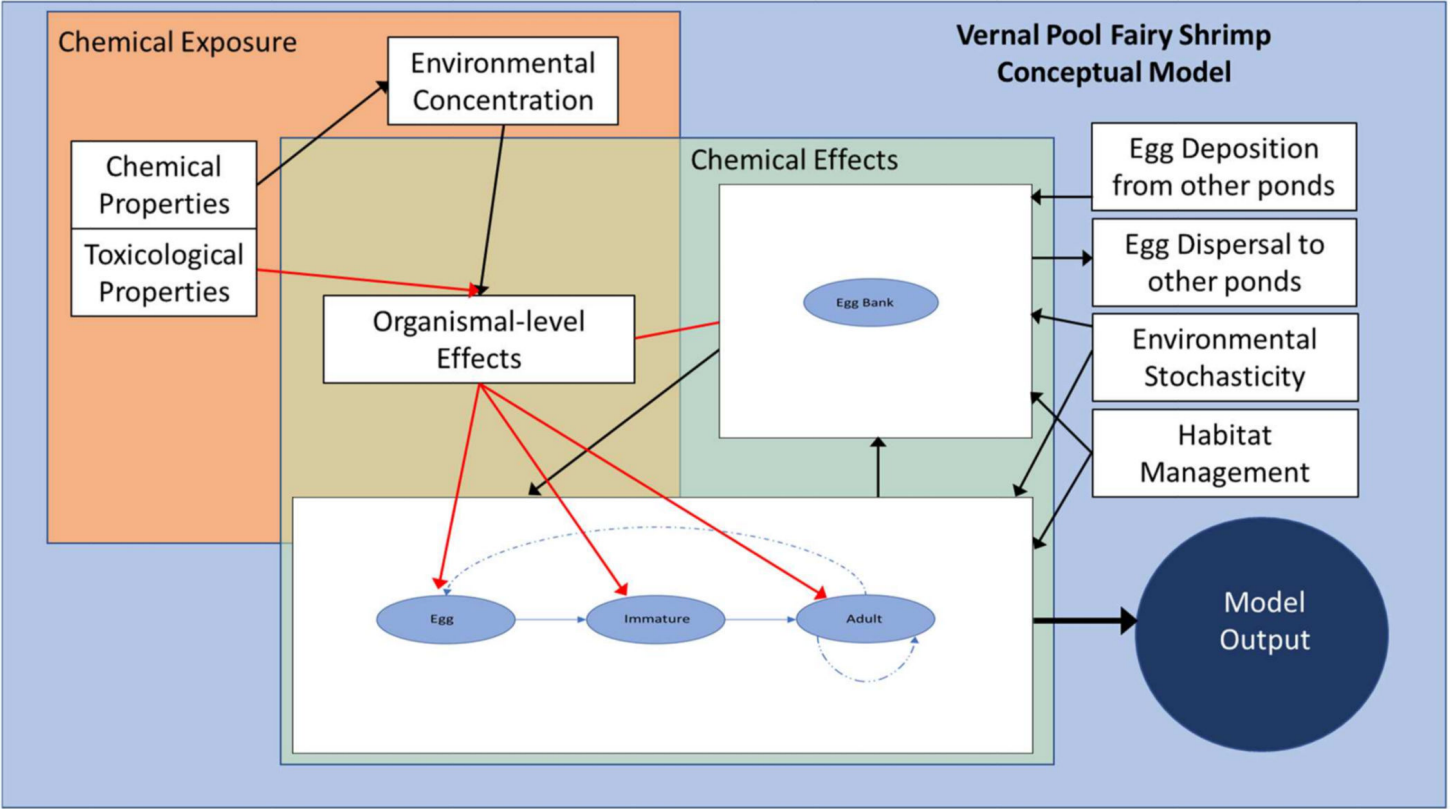
A conceptual model demonstrating both an illustrational and written summary of the compartments within a vernal pool fairy shrimp population model and their linkages. The egg bank comprises eggs accumulated in the substrate over a multiple year period. The red arrows depict adverse pathways associated with a pesticide, and all other connections are represented by black arrows.

A conceptualization demonstrating both an illustrational and written summary of the compartments within a vernal pool fairy shrimp population model and their linkages was derived (Figure 3). The aggregate of the modeling space (identified by the gray box) contains overlapping subcomponents for chemical exposure (identified by the orange box) and chemical effects (identified by the green box). Model components are represented by white boxes and their connections are represented by arrows. The red arrows depict adverse pathways associated with a pesticide, and all other connections are represented by black arrows. Environmental stochasticity was considered independent of exposure and effects. The extent for overlap of various components is conceptual and is qualitative as opposed to a scaled quantitative representation of overlapping layers.

Within the space identified as chemical exposure, chemical and toxicological properties would be used to inform environmental concentrations (i.e., through the use of exposure models such as PWC). The model component identified as organism-level effects on vernal pool fairy shrimp would be informed by effects measured in laboratory studies (currently available laboratory studies used surrogate species) and driven by concentrations estimated to be in the field. Chemical exposure and effects would be used to inform dynamics of all stages as depicted by the life cycle diagram (e.g., immature and adult stages) and may also impact the egg bank (i.e., survival of eggs accumulated in the substrate over a multiple year period). In addition, since the population in a given vernal pool can be very small, environmental stochasticity was included to account for extrinsic influences (see Figure 3). Furthermore, as *B. lynchi*, are federally listed as threatened, management activities such as monitoring and recovery initiatives may inform demographic rates (represented by the “Habitat Management” compartment in Figure 3).

#### 3.1.5. Phase 5: Evaluation and implementation

We utilized the data collection (Tables S1–S4), and the decision steps phases (see Figure S1, Supplemental Materials) to examine how the conceptual model (Figure 3) could be implemented within the context of a risk assessment for *B. lynchi* within Central Valley, California. While data were gathered from multiple study sites across California (Tables S1–S4), a model developed for Merced County agricultural area of California’s Central Valley would allow for application of the PWC specific to interactions among vernal pools located in the San Joaquin River basin in the Central Valley of California. This would enable time series of temporal and spatial surface water and sediment pore water pesticide concentrations for diazinon and malathion specific to given vernal pool watersheds [8]. Modeled vernal pools could provide EEC profiles representative of the diverse VPs in California for broader applications. In addition, PWC is capable of simulating dynamic complex vernal pool hydrology including estimation of volume and depth. Finally, daily temperature is included as an input required by PWC’s hydrologic component, allowing for a time series of temperature specific to any given vernal pools to be generated [8].

Since their introduction over 50 years ago, structured population models have become a central modeling formalism, and one of the most commonly used in ecology [58,59]. The use of matrix population models within ecological risk assessments is advantageous because such models integrate effects across the life cycle, link endpoints observed in the individual to ecological risk for the population as a whole, and project outcomes over future timespans allowing for management planning scenarios. Matrix models have been used to investigate population dynamics of other Anostracans [24,43,44], and could be applied to vernal pool fairy shrimp. Vital rates of survivorship and fertility would be defined using functions (i.e., a quantitative relationship between fecundity and temp and application of survival rates that vary with temperature but also incorporate a threshold temperature). The projection matrix could be separated into a matrix of fertility rates and a matrix of survival rates to allow for egg production in a given year to contribute solely to the egg bank and to be exclusive and prohibited from contributing to the current season’s immature and adult organism count [60].

In moving beyond the conceptual model to implementation of a quantitative population model, some recommendations for evaluation are perceptible. In considering the use of PWC estimates, validation of vernal pool depths with observed data would help calibrate the vernal pool dynamics of the model and improve exposure concentration predictions. In addition, life history characteristic that impact vernal pool fairy shrimp dynamics (i.e., hatchability of eggs) are also influenced by hydroperiod length. Empirical data that could be compared to model outputs for verification could include measurement of chemical concentrations at the vernal pool site following major precipitation. For example, Johnson (2006) measured diazinon concentration from three vernal pools on the Kesterson National Wildlife Refuge within 24 h of a major storm event [7]. Additionally, measurements of vernal pool fairy shrimp reproduction and survival, as well as estimates of population size and longevity at both reference and exposed vernal pool sites coordinated with measurements in weather patterns over time would be desirable for comparison to model outputs.

## 4. Discussion

California contains 24 species of fairy shrimp constituting 47% of the entire fairy shrimp fauna of North America, including 7 species that are found nowhere else in the world [48,61]. It is estimated that between 60% to 85% of temporary waters in the California Central Valley have been lost to development and human activities [5,48]. One example of human activities is agriculture, and the impact of organophosphate pesticides on vernal pool fairy shrimp population status was investigated as a test case in the present study. As *B. lynchi* is currently listed as threatened, understanding the ecological risks posed to vernal pool fairy shrimp is important for conserving the existing populations in California from further decline, as well as protecting vernal pool critical habitat.

Population models offer the ability to translate organism-level endpoints into effects on the population and have the potential to incorporate multiple stressors (both biotic and abiotic) and landscape attributes. Thus, population models provide a tool that can be used in ecological risk assessments to aid in understanding population response to management actions and for development of recovery plans. In the present study, Pop-GUIDE was employed to facilitate development of a population model that could be used to examine effects of organophosphate pesticides on vernal pool fairy shrimp. We demonstrated how Pop-GUIDE provided a systematic approach to the process of model development. We used accessible data for vernal pools in Central Valley, California employing a multistep process that identified model objectives and data availability. Based on ecosystem, chemicals, and endpoints of interest to the assessment objectives, a conceptual model was developed commensurate with the quality and quantity of data available. The materials created through the execution of the sequential phases provided documentation of the model building process. Thus, using this approach resulted in production of a record of the conceptual model development and illustrated how Pop-GUIDE could be used as a framework suitable for transparently conveying a population model to stakeholders.

**Figure 4.**
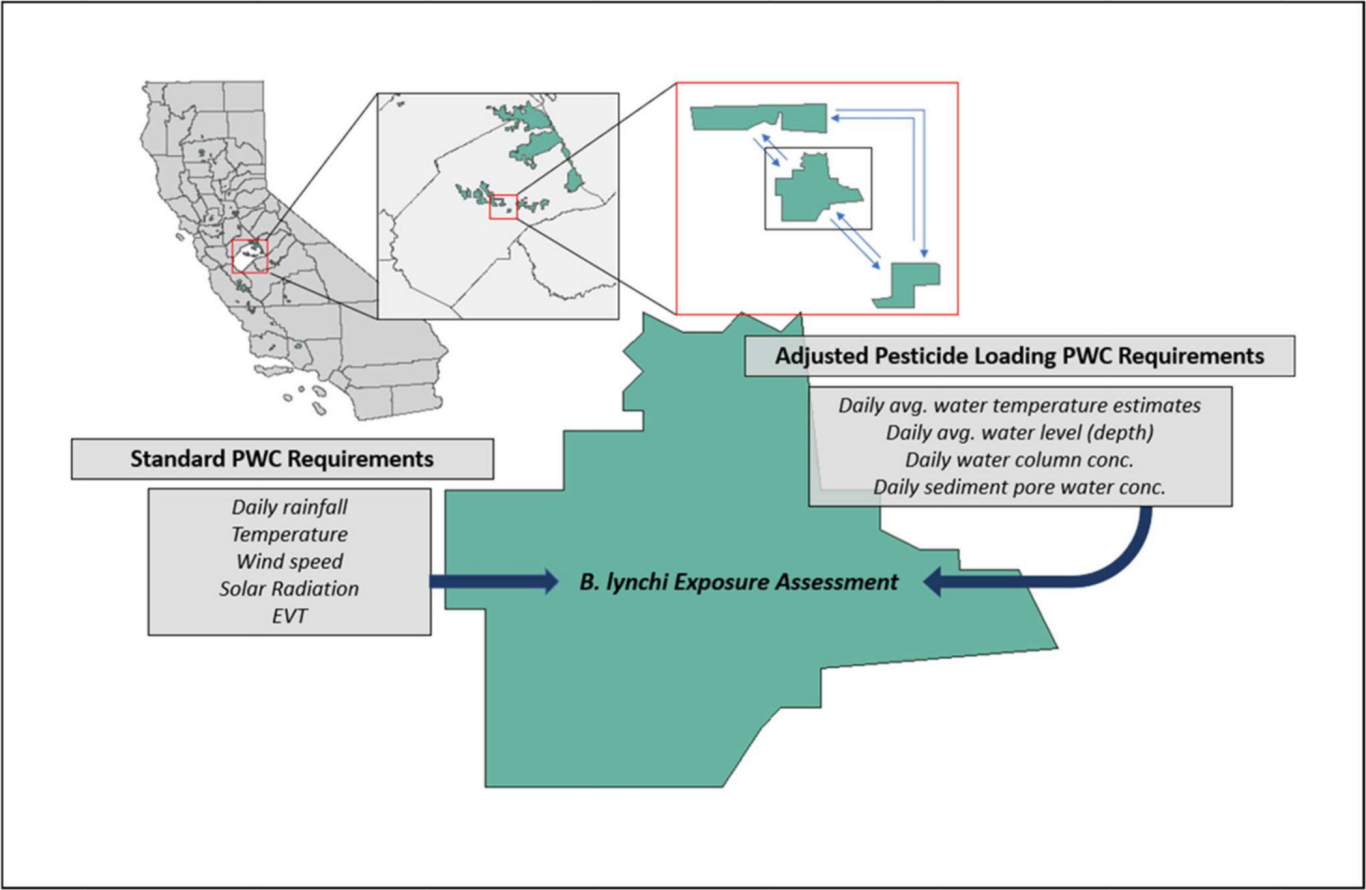
Standard and adjusted Pesticide in Water Calculator (PWC) parameters required for estimating exposure concentrations for diazinon and malathion, along with locations of vernal pool critical habitat for *B. lynchi* in California, specifically several pools within Merced County as an example. Note that exposure effects for individual pools will impact dynamics between adjacent vernal pools.

In Phase 1, we decided that the model objective was to provide reliable estimates of potential impacts of realistic pesticide exposure scenarios on vernal pool fairy shrimp abundance using available data at a specific vernal pool location in California’s Central Valley. The data collection process (Phase 2, Table S4) identified that PWC had been used previously to predict surface water and sediment pore water concentrations for diazinon and malathion in vernal pool sites in the San Joaquin River basin [8]. These sites are within the Merced County agricultural area of California’s Central Valley, which ranks high in pesticide application rate, falling sixth and seventh among 58 counties in California in 2014 and 2015, respectively [54] based on total pounds of active ingredients applied [8,54]. The adjusted PWC output could be utilized to build a *B. lynchi* exposure assessment for a given vernal pool (Figure 4), and incorporated as part of a larger metapopulation analysis to evaluate population dynamics for *B. lynchi* across multiple pools. Exposure effects that potentially reduce fairy shrimp abundance within individual pools may impact dynamics between adjacent vernal pools resulting from a reduction in eggs capable of being dispersed to adjacent pools.

The combination of landscape scale analysis such as that provided herein by PWC coupled with a population model for vernal pool fairy shrimp could be transferable to other areas and used to ‘scale up’ to include ponds/populations at locations in California whereby the appropriate data (i.e., crop and pesticide, weather, elevation, soil properties) are available for input. In a broader context, the results of variable exposure concentrations of pesticides could be explored over a distribution of vernal pool sites, with a focus on how varied exposure concentrations could lead to accentuated changes in vernal pool fairy shrimp abundance. In scaling the model to consider the context of a response of metapopulations to chemical exposure, the egg bank (see Figure 3) may play an important role in population sustainability.

While the population model developed herein focused on organophosphate pesticides, alternative scenarios could also evaluate other chemical stressors. The University of California, Davis, as commissioned by the California Central Valley Regional Water Quality Control Board (CVRWQCB) has derived aquatic life criteria for several pesticides of particular concern in the Sacramento River and San Joaquin River watersheds, which are also widely used throughout the USA. In addition to organophosphate insecticides, pyrethroid insecticides including bifenthrin, cyfluthrin, cypermethrin, lambda-cyhalothrin, and permethrin, as well as the phenyl-urea herbicide diuron have been evaluated by the University of California, Davis [52]. The population model developed for vernal pool fairy shrimp could be used in combination with PWC to investigate potential impacts to these additional agricultural pesticides.

Climate changes, including increased temperatures and increased frequency of extreme weather events, have and will continue to impact water quality and water supply throughout California, which will in turn affect vernal pools. Within the Central Valley, greenhouse gas emissions scenarios predict a median increase in annual temperature of approximately 2 °F by 2025 and 4 °F by 2060 and heat waves are also expected to increase in frequency, with individual heat waves also showing a tendency towards becoming longer and extending over a larger area [62]. Recent studies suggest that the impact to vernal pools from increased warming can range from decreased duration of the wet phase to alterations in species composition [63,64]. Precipitation rate projections also indicate impending changes, as 11 of the 12 precipitation models run by the Scripps Institution of Oceanography suggest a 12% to 35% overall decrease in precipitation levels by mid-century, which would have additional consequences for the hydrology and composition of vernal pools [62,65]. In the present study, data collection in Phase 2 revealed that several life history characteristics are driven by environmental factors and will be affected by changes in temperature and/or rainfall. For example, maturation, reproductive frequency and reproductive output are all influenced by temperature (Phase 2, Table S1). Both temperature and pool inundation affect hatching of eggs, as well as survival rates and persistence of the population (Phase 2, Table S1) [34,35,41,42]. Global climate change resulting in alterations in temperature and rainfall patterns will also impact the magnitude and time series associated with pesticide exposure at a given vernal pool location. Modeling climate change can be incorporated by adjusting the corresponding weather inputs required by PWC’s hydrologic component (i.e., daily rainfall, temperature, wind speed, solar radiation, and evapotranspiration) as well as through functions that link survival, reproduction, and hatching to temperature and rainfall.

Increased global temperature and CO_2_ levels and regionally specific changes in precipitation patterns are also predicted to impact pesticide application rates [14,66]. Range expansion and conditions that potentially increase pest pressure could lead to increased pesticide application, as could a longer growing season and enhanced plant growth due to increased CO_2_ [13]. Predictions of climate change impacts on fairy shrimp habitat and pesticide application are variable and likely to depend upon interactive effects of climate change on temperature, precipitation, crop type and pest responses. Potential scenarios based on climate-related predictions are important components of a population model applied to ERA for the threatened vernal pool fairy shrimp, a species vulnerable to multiple climate stressors impacting critical habitat and altering pesticide application.

## 5. Conclusions

We utilized Pop-GUIDE as a comprehensive tool to provide a systematic process of conceptual model development using a standardized series of phases and to serve as documentation of the data collection and decision steps taken in model formulation, thereby facilitating subsequent model implementation and evaluation. In summary, our population model demonstrates the use of Pop-GUIDE for a threatened species to estimate risks of long-term impacts to vernal pool critical habitat from exposure to multiple stressors (both biotic and abiotic). Using the different phases of Pop-GUIDE, we can infer how stressors can influence *B. lynchi* populations either singly or in multiple stressor scenarios. The model translates impacts on individuals into changes at the population level, and allows simulation of a number of exposure scenarios across their distributed range in the Central Valley of California. The present analysis provides a case study that illustrates the value of implementing Pop-GUIDE as an approach that could be administered to support ecological risk assessment and advance conservation planning for threatened and endangered species.

## Supplementary Material

Table S1

Table S2

S1

Table S3

Table S4

## Figures and Tables

**Table 1.** Phase 1 questions to derive model objectives.

Number *	Question
1.	Ideally, how will the population model be used in the ERA, e.g., as a direct assessment tool in species- or location-specific ERA (e.g., endangered species, superfund) or as part of a weight of evidence for broader ecological protections? If the model will be used as a direct assessment tool, its tradeoffs should match that of the ERA category.
2.	What assessment endpoint(s) are most relevant to the ERA objective and the intended model use (e.g., population growth, abundance, quasi-extinction probability, etc.)?
3.	Are there temporal considerations that are important to the realism of the ERA, e.g., seasonal chemical application or persistence in the environment?
4.	What uncertainties are acceptable for the ERA?
5.	What are the project resources (timeline, budget, etc.)?

* question numbers correspond to those from Box 1., Phase 1. In Pop-GUIDE [28].

## Data Availability

All data produced from this study are provided in this manuscript.
