# Supplementary material for: Investigating Vernal Pool Fairy Shrimp Exposure to Organophosphate Pesticides: Implications for Population-Level Risk Assessment
Source: Ecologies (Basel). Author manuscript; Available in PMC 2023 Aug 2. (PMC9769362; doi:10.3390/ecologies3030024)
Supplement: Table S1 [file NIHMS1829936-supplement-Table_S1.pdf]

Table S1. , Supplementary Materials. Organism-level characteristics.

Characteristic	General	Realistic	Precise	Taxonomic Specificity	Reference
Life span		Maximum observed lifespan for egg stage is several years.	Maximum observed lifespan for adult stage is of 5 to 7 weeks.	*Branchinecta lynchi*, Branchiopoda	[31,32,34]
Reproductive frequency		Reproductive period observed to last up to 4 weeks depending upon duration of pool inundation (pool depth and duration), with a longer duration of pool inundation correlated with a longer reproduction period.		*Branchinecta lynchi*	[34]
Reproductive output/clutch size		*Branchinecta* were observed to have a maximum number of eggs per clutch of 67. *Branchinecta lindahli* were found to have an average clutch size from 3.25 to 52.5 that varied by location and with temperature.	Cultured *Branchinecta lynchi* were found to have a total clutch of 9 to 126 that varied with temperature.	*Branchinecta* sp., *Branchinecta lindahli*, *Branchinecta lynchi*	[34,35]
Onset of maturation		Females observed with eggs at 2 to 4 weeks, with quicker onset of maturation occuring at pools that were sampled later in the season at higher temperatures.	First Clutch observed at 6d Palo Cedro, Shasta Co. California, June 1993, Size of 2. First Clutch observed at 12d, Tehama Co. California, January 1988, Size of 24.	*Branchinecta lynchi*	[34,35]
Hatching (i.e. eggs/female/d)		Hatching of eggs dependent upon temperature and vernal pool water level. Depending on species (genetics) and population (epigenetics), typical hatching fractions will vary between 0.2 and 70%.		*Branchinecta lynchi*, Branchiopods	[31,34,36-40]

Immature transition rate (including metamorphosis)[1]		*Branchinecta* sp. was observed to have a transition occuring at day 12 post hatch of 40% Adult; 60% immature. No immature found at sampling 21d post hatch.		*Branchinecta* sp.	[41]
Sex ratio		*Branchinecta* sp. was observed to have a statistically significant equal sex ratio using observations taken at a field site using observations collected over multiple years.		*Branchinecta* sp.	[41]
Recruitment rate			*Branchinecta lynchi* was observed to have up to 3 distinct hatches (egg emergence) in 1 season which were dependent on pools drying and refilling with cold temperature water.	*Branchinecta lynchi*	[34]
Survival rate		The fairy shrimp, *Phallocryptus spinosa* had a cumulative survival measured at 15C of 99% at day 3, 92.3% at day 6, 88% at day 9, 86.1% at day 12, 82% at day 15 and 78.8% at day 18. Daily survival ranged from 97.7% to 99.3%.		*Phallocryptus spinosa*	[42]
Growth rate			Maximum size of 1 inch. Ranges in size from 10.9 to 25.0 mm (0.4 to 1.0 inches).	*Branchinecta lynchi*	[5,34]
Egg Emergence		Vernal pool fairy shrimp have been collected from early December to early May.	Hatched juvenile fairy shrimps were first observed two weeks after the pools filled with standing water.	*Branchinecta lynchi*, *Branchinecta* sp.	[5,34,41]

			Hatching first observed when pond reached 11-20cm of water depth.		
Egg Dormancy Duration		When pools refill in the same or subsequent seasons some, but not all, of the eggs may hatch. The egg bank in the soil may be comprised of the eggs from several years of breeding. Depending upon Anostracan species and population, typical hatching fractions will vary between 2% and 70%.		*Branchinecta lynchi*, *Anostracan* sp.	[5,31,32]

[a.] In Gallagher (1996), distribution and occurrence of *Branchinecta lynchi* in a large vernal-pool complex in Butte County, California, U.S.A., were sampled. The 105 ha vernal-pool complex was located northeast of the City of Chico, California (T 22 N, R 2 E, Sec 22), at an elevation of 60m [34].

[b.] In Patton (1984), the life history patterns of *Branchinecta* sp. were observed in a temporary pond located 25.6 km north of Chico, California and a series of similar ponds located over a 66km stretch of landscape (43 ponds located from Richvale vernal pools to Vina Plains) [41].

[c.] In Rogers (2015), responses in *Branchinecta lindahli* cultures collected from four populations across 8° of latitude in Lassen County, Riverside County, San Luis Obispo County and Yolo County, California, USA were investigated [35].

[d.] In Patton (1984), in examining sex ratio, the analysis was done using a Chi-Square test over multiple seasons, Chi-Square = 1.44, $p = 0.05$ [41].

[e.] A cell that is left blank reflects that no data was collected.
