# Supplementary material for: Investigating Vernal Pool Fairy Shrimp Exposure to Organophosphate Pesticides: Implications for Population-Level Risk Assessment
Source: Ecologies (Basel). Author manuscript; Available in PMC 2023 Aug 2. (PMC9769362; doi:10.3390/ecologies3030024)
Supplement: Table S2 [file NIHMS1829936-supplement-Table_S2.pdf]

Table S2. Supplementary Materials. Population and spatial characteristics.

Characteristic	General	Realistic	Precise	Taxonomic Specificity	Reference
Density dependence	Logistic density dependence used to model Crustacean population growth.	Impairment and population decline observed with increased density in Mysidopsis species. Higher densities of individuals per litre result in decreased fitness.	Maximum population density of 200 individuals/liter water. Observation of 0.33 to 23.67 individuals/cubic meter.	Mysid shrimp (*Americamysis bahia*), Mysidopsis species, *Branchinecta lynchi*, *Branchinecta lindahli*	[24,35,43-47]
Population size			Physical characteristics (temperature and pool inundation) impacted both the distribution and number of vernal pool fairy shrimp sampled.	*Branchinecta lynchi*	[34]
Spatial metapopulation structure		This species has a sporadic distribution within vernal pool complexes wherein the majority of pools in a given complex may not have the species observed in them. In a given location, a population may be comprised of a single inhabited pool within a complex.		*Branchinecta lynchi*	[1,5]
Movement		Mammals, birds, fish, amphibians, crayfish, and insects provide a "directed" passive dispersal vector whereas wind provides a "random" passive dispersal vector.		*Branchinecta lynchi*	[31,32]
Habitat features		Observed Temperature Range of -7.8c to 32C for *Branchinecta lindahli*	Habitat duration of between 3 and 14 weeks.	*Branchinecta lynchi*, *Branchinecta lindahli*	[34,35]
Geographical range	Endemic to vernal pools in the Central Valley, coast ranges, and a limited number of sites in the Transverse Range and		Three vernal pool sites located in Merced County agricultural area of California's Central Valley. Sites include Canal Creek Tributary Watershed site,	*Branchinecta lynchi*	[5,8]

	Santa Rosa Plateau of California.		Owens Creek Tributary Watershed site, and Middle Mariposa Slough Tributary Watershed site.		
Habitat classification/suitability			Mean pool area of pools containing *Branchinecta lynchi* was 147.1 meters squared.<br>Mean pool depth of pools containing *Branchinecta lynchi* was 0.14m.<br>Mean pool volume of pools containing *Branchinecta lynchi* was 20.3 meters cubed.	*Branchinecta lynchi*	[34]

[a.] The density dependent factor used in Thursby (2016) and Thursby et al., (2018) is calculated based upon the formulation first described by Leslie, 1948 [24,43].

[b.] In Gallagher (1996), distribution and occurrence of *Branchinecta lynchi* in a large vernal-pool complex in Butte County, California, U.S.A., were sampled. The 105 ha vernal-pool complex was located northeast of the City of Chico, California (T 22 N, R 2 E, Sec 22), at an elevation of 60m [34].

[c.] In Rogers (2015), responses in *Branchinecta lindahli* cultures collected from four populations across 8° of latitude in Lassen County, Riverside County, San Luis Obispo County and Yolo County, California, USA were investigated [35].

[d.] In Sinnathamby et al. (2020), Figure 1., a map of the state of California illustrating the spatial location of each of three vernal pool sites, can be found [8].

[e.] A cell that is left blank reflects that no data was collected.
