# Supplementary material for: Investigating Vernal Pool Fairy Shrimp Exposure to Organophosphate Pesticides: Implications for Population-Level Risk Assessment
Source: Ecologies (Basel). Author manuscript; Available in PMC 2023 Aug 2. (PMC9769362; doi:10.3390/ecologies3030024)
Supplement: S1 [file NIHMS1829936-supplement-S1.pdf]

Figure S1, Supplemental Materials: Decision Steps Key: Panel A: II.) Organism-level processes, Panel B.: III.) Population and spatial factors, Panel C.: IV.) External factors, Panel D.: V.) Exposure and effects. Decisions made for each question are highlighted in yellow.

2.1 Growth and development

	Question	Yes	No
1	Is there sufficient information to represent growth continuously?	2	No additions to model concept
2	Is there sufficient information to represent growth physiologically?	Physiologically-based (e.g. DEB, other physiological approaches) representation of growth	Simple growth model may be included (Von Bertalanffy, Gompertz, Logistic, other)

2.2 Maturation and reproduction

	Question	Yes	No
	Is there sufficient information to represent maturation dependent on body size /mass/energy reserves?	Maturation dependent on body size /mass/energy reserves	Maturation dependent on age (constant or distribution)
	Is there sufficient information to represent fecundity dependent on body size/ mass/ energy reserves or specific age?	Fecundity dependent on body-size/ mass/ energy reserves or age	Fecundity constant or from a distribution

Figure S1., Supplemental Materials, Panel A.

3.1 Population Status

	Question	Yes	No
1	Is the estimated species' overall abundance or population size critically small?	Represent environmental stochasticity as well as variation in demographic rates	2
2	Are data available to demonstrate that populations exhibit high variability between years, i.e. do the population dynamics considerably deviate from stability?	Represent variability through environmental stochasticity and demographic variance (if available)	Variation can be represented by demographic factors alone

3.2 Density dependence

	Question	Yes	No
1	Do populations experience documented resource or space limitation?	2	Negative density dependence does not need to be considered. Continue to 5.
2	Are the factors driving density dependence (e.g., spawning sites, food limitations, shelter) known?	3	Apply ceiling type density dependence
3	Does density affect individuals in the population differently dependent on their life stages or size, or only affect processes unique to a single life stage?	4	Implement a consistent density dependence survival function (e.g., Ricker) across all life stages
4	Does population density affect growth or reproduction?	Implement life-stage specific density dependence, either emergent or imposed, on growth/fecundity	Represent density dependence as survival function for appropriate life stage(s).
5	Are populations reported to experience Allee effects due to low densities?	Include Allee effect, e.g., as function linking fecundity to adult abundance	No additions to model concept

3.3 Movement

	Question	Yes	No
1	Do individuals migrate/disperse outside the action area to complete their life cycle?	Incorporate function of migration/ dispersal to represent time spent in action area, as well as important movement within action area	2
2	Is migration/ dispersal of individuals important, and may interact with exposure?	3	Migration/dispersal does not need to be represented
3	Do the individuals systematically move across habitat types and/or metapopulation patches in response to resource availability, predation pressure, or migration/dispersal to natal habitat?	Incorporate function of migration/ dispersal in model; represent metapopulation dynamics as appropriate	Migration/dispersal does not need to be represented; model focuses on single habitat type

3.4 Behavior

	Question	Yes	No
1	Are aspects of behavior *other than* migration/dispersal known to be affected by exposures?	2	No additions to model concept
2	Are data available to mechanistically/explicitly represent behaviors potentially affected by exposure?	Include explicit representation of identified behaviors	3
3	Can effects on behavior be linked to submodels (e.g., survival, growth, bioenergetics, fecundity)?	Represent effects on behavior as impacts on appropriate submodel	Categorical or qualitative impacts on behavior could be included to add realism

3.5 Habitat features

	Question	Yes	No
1	Does the species occupy/use more than one habitat type that needs to be represented in the model (e.g., different exposures	2	No explicit representation of space

	occurring in the habitats, and are data available to distinguish between habitats)?		in the model
2	Are data available to distinguish between habitats?	3	Spatially-implicit representation
3	Are spatially explicit interactions with the habitat defined?	Explicitly represent spatial distributions of habitat and/or exposure	Represent habitat(s) implicitly, e.g., along a single axis (e.g., location in the water column, stream section)

Figure S1., Supplemental Materials, Panel B.

4.1 Diet

	Question	Yes	No
1	Is the species' diet expected to be impacted by the chemical (based on mode of action or available data)?	2	No additions to model concept
2	Are data available to determine how the species' diet may be affected by the chemical exposure (e.g., dose-dependent food reduction)?	3	Categorical or qualitative impacts to diet could be included
3	Can impacts on prey be represented as effects on vital rates (e.g., growth, survival)?	4	Quantitative impacts to diet could be included
4	Is there enough information to represent the species diet explicitly and/or temporally/spatially varying?	Represent the diet as a time-series of separate or aggregated resource items.	Include indirect effects mediated by diet as impacts on appropriate vital rate estimates

4.2 Other interspecific interactions

	Question	Yes	No
	Does the species have obligatory relationships with other species potentially affected by the chemical?	Include indirect effects mediated by obligatory relationship as impacts on appropriate submodel(s)	No additions to model concept
	Are biota critical to habitat integrity potentially affected by the chemical stressor (e.g., cover from predators/herbivores)?	Consideration of inclusion if indicated that the species is strongly dependent on specific conditions that may be affected by the stressor	No additions to model concept

4.3 Abiotic factors

	Question	Yes	No

	Are environmental conditions indicated to be important drivers of the population dynamics (e.g. temperature, precipitation, water depth, stream flow, flood events, habitat connectedness, etc.) and do those drivers differ between years or habitats of the species?	Dependence on environmental condition should be represented; Variation in across-year environmental conditions may be captured by stochasticity in the model	Species-specific environmental conditions do not need to be represented explicitly in the model
	Is the species impacted by additional stressors not previously discussed that may interact with the chemical stressor?	Include relationships as impacts on appropriate submodel(s)	No additions to model concept
	Is the species the subject of an existing management plan that may influence exposure probability or effects?	Include relevant management scenarios in model, as appropriate	No additions to model concept

Figure S1., Supplemental Materials, Panel C.

5.1 Exposure

	Question	Yes	No
1	Do multiple exposure routes need to be considered, e.g., contact exposure, dietary exposure, exposure via nesting material?	2	Include exposure via primary route as measured in tests
2	Are separate sets of toxicity data available to inform the effects from each exposure route?	Represent each exposure route separately	Combine the exposures from the exposure routes for the effect representation

5.2 Mortality effects

	Question	Yes	No
1	Are measured effects on survival available?	2	No direct effects on survival represented in the model
2	Are lethal effects endpoints available from multiple studies with different durations, e.g., acute and chronic studies?	3	If acute data only are available, go to 3;<br>If chronic data only are available, go to 4
3	Can the available acute data be used to fit a time-variable effects model, e.g. toxicokinetic-toxicodynamic model (TKTD / GUTS)?	Represent effects of time-variable exposures based on all study data; continue to 4	4
4	Are chronic survival data available for all concentrations?	Represent survival effects as dose-response functions or TKTD models	Represent survival effects as a threshold (e.g., NOEC/LOEC)

5.3 Sublethal effects

	Question	Yes	No
1	Are data on sublethal effects available, e.g., effects on growth, reproductive rate, etc.?	2	Sublethal effects are not represented in the model

2	Can measured sublethal effects be linked with corresponding organism-level processes explicitly represented in the model, e.g., bioenergetics?	Sublethal effects represented affecting the corresponding organism-level process via e.g. dose-response or TKTD models	3
3	Can sublethal effects be expressed as reduction in growth, reproductive, and/or population survival rates?	'Translate' sublethal effects to lethal effects and/or impacts on growth and/or fecundity	Sublethal effects are not represented in the model

5.4 Temporal representation

	Question	Yes	No
1	Are important toxicological processes represented at fine resolution (e.g., TKTD processes)?	Time step consistent with temporal resolution of process with shortest time step; or nest functions with shorter time steps within larger time step.	2
2	Are seasonal differences important to the species' life cycle and need to be considered to adequately describe species and chemical co-occurrence?	Temporal resolution < 1 year reflecting temporal scale of seasonal changes	Default to annual time step

Figure S1., Supplemental Materials, Panel D.
