# Supplementary material for: Investigating Vernal Pool Fairy Shrimp Exposure to Organophosphate Pesticides: Implications for Population-Level Risk Assessment
Source: Ecologies (Basel). Author manuscript; Available in PMC 2023 Aug 2. (PMC9769362; doi:10.3390/ecologies3030024)
Supplement: Table S3 [file NIHMS1829936-supplement-Table_S3.pdf]

Table S3. Supplementary Materials. External factors.

Characteristic	General	Realistic	Precise	Taxonomic Specificity	Reference
Predation		Aquatic predatory water birds, amphibians, and insects provide a "directed" passive dispersal vector (for eggs).<br>Fairy shrimp are fed upon by waterfowl and other vertebrates, such as western spadefoot toad (*Scaphiopus hammondi*).<br>Fairy shrimp feed on algae, bacteria, protozoa, rotifers, and bits of detritus.		*Branchinecta lynchi*	[5,31,32]
Competition	Only rarely does the vernal pool fairy shrimp co-occur with other fairy shrimp species, but where it does,<br>the vernal pool fairy shrimp is never the numerically dominant one.	Has been observed in pools with *Linderiella occidentalis* and *Lepidurus packardi*. These two species were observed to be more tolerant of high temperature and low oxygen than *Branchinecta* lynchi. Has also been observed alone.<br>Has rarely been observed in pools with *B. mackini, B. lindahli, B. longiantenna, and B. conservatio.*		*Branchinecta lynchi*	[1,5,34,48]
Environmental conditions		Duration of pool inundation (pool depth and duration)<br>are important to the occurrence of *Branchinecta lynchi*.	Observations of *Branchinecta lynchi* involved locations where the recorded temperature range was between -5C and 25C.	*Branchinecta lynchi*	[5,34]
Stressors - pathogens	N/A	N/A	N/A	N/A	N/A
Stressors – abiotic, other		In danger of extinction principally as the result of urban development, conversion of native habitats to agriculture,		*Branchinecta lynchi*	[5]

		and stochastic (random) extinction by virtue of the small isolated nature of many of the remaining populations.			
Existing management			Listed as a vulnerable species on the IUCN Red List. Listed under the U.S.A. Federal Endangered Species Act, September 19,1994.	*Branchinecta lynchi*	[5,49]
Indirect effects (obligatory relationships)	N/A	N/A	N/A	N/A	N/A

[a.] "N/A" is used to indicate "Not Applicable".

[b.] In Gallagher (1996), distribution and occurrence of *Branchinecta lynchi* in a large vernal-pool complex in Butte County, California, U.S.A., were sampled. The 105 ha vernal-pool complex was located northeast of the City of Chico, California (T 22 N, R 2 E, Sec 22), at an elevation of 60m [34].

[c.] A cell that is left blank reflects that no data was collected.
