# Supplementary material for: Investigating Vernal Pool Fairy Shrimp Exposure to Organophosphate Pesticides: Implications for Population-Level Risk Assessment
Source: Ecologies (Basel). Author manuscript; Available in PMC 2023 Aug 2. (PMC9769362; doi:10.3390/ecologies3030024)
Supplement: Table S4 [file NIHMS1829936-supplement-Table_S4.pdf]

Table S4., Supplementary Materials. Exposure and effects characteristics.

Characteristic	General	Realistic	Precise	Taxonomic/Location Specificity	Reference
Chemical exposure		PWC version 1.59 has been used to estimate daily pesticide concentrations of Diazinon and Malathion in vernal pools in Central Valley, California.	Diazinon concentrations were measured at three vernal pool sites within the San Luis National Wildlife Refuge in the San Joaquin Valley of California during 2002-2003.	San Luis Wildlife Refuge, San Joaquin Valley, California and Merced County agricultural area of California's Central Valley	[7,8,51]
Temporal exposure pattern		Using PWC, Pesticide application rate and timing were inferred based on the Merced County, Public Land Survey System (PLSS) Pesticide Use Reporting (PUR) database from California Department of Pesticide Regulation (CDPR). The PLSS data set includes Township, Range, and Section land parcels in State of California.		Merced County agricultural area of California's Central Valley	[8,51]
Exposure pattern within and across habitat		Using PWC, and based upon physiochemical properties, application of organophosphates including diazinon and malathion were modeled spatially for vernal pools in Merced County agricultural area of California's Central Valley.<br>Diazinon was applied as a dormant season application, and malathion was applied aerially. NASS Cropland Data Layer (CDL) 2007 crop coverages to generate spatially-relevant aquatic exposure concentrations.		Merced County agricultural area of California's Central Valley	[8,51]
Representation of toxic effects	California Regional Waterboard aquatic life criteria, pesticide threshold concentrations for aquatic invertebrates,	Web-ICE predictions for vernal pool fairy shrimp threshold concentration:<br><br>3.64 ppb (0.95-14.02)<br>Diazinon		*Thamnocephalus platyurus*, *Daphnia magna*,<br>Collection of species including crustaceans	Oliver and McCaffrey, Unpublished laboratory data, [8,52,53]

	Diazinon: Acute 0.2 ug/l, Chronic 0.2 ug/l Malathion: Acute 0.17 ug/l, Chronic 0.28 ug/l<br><br>ECOTOXicology Knowledgebase System, data for various pesticides	6.64 ppb (1.87-23.52) Malathion<br><br>1. 24h LC50 0.14±0.04, 2.28±0.11 mg/l Diazinon<br><br>39.46±1.54, 21.68±1.11 mg/l Malathion			
Effects by life stage or size		nstar nauplii		*Thamnocephalus platyurus*	Oliver and McCaffrey, Unpublished laboratory data
Effects depending on exposure route (e.g. dietary, chemical in water)		All aqueous exposures		*Thamnocephalus platyurus*	Oliver and McCaffrey, Unpublished laboratory data

[a.] Pesticide in Water Calculator (PWC) is a USEPA model that simulates surface and groundwater pesticide concentrations resulting from land application [51].

[b.] The Public Land Survey System (PLSS) Pesticide Use Reporting (PUR) database from the California Department of Pesticide Regulation (CDPR) includes Township, Range, and Section land parcels in the State of California and annually reports agricultural pesticide use at PLSS one-square mile sections and non-agricultural pesticide use by active ingredient at county level (for further information see CDPR, (2018, [54]).

[c.] The organophosphates Diazinon and Malathion are commonly used in California's Central Valley, and detected in snow, air and surface waters in the mg/L or mg/L levels (for further information see [15-18,55,56].

[d.] Web-ICE is the Web-based Interspecies Correlation Estimation application:

(Web-ICE; https://www3.epa.gov/ceampubl/fchain/webice/; accessed 10/23/18; for more information see Raimondo et al., (2015) and Raimondo et al. (2019) [4,19]. Web-ICE predictions were evaluated using the criteria recommended by Willming et al. (2016), [57]. The Web-ICE predictions for vernal pool fairy shrimp threshold concentration used the following surrogate species: Evaluation of Diazinon, surrogate species used was *Daphnia magna* (1.9 mg/L); Evaluation of Malathion, surrogate species used was *Daphnia magna* (3.7 mg/L).

[e.] A cell that is left blank reflects that no data was collected.
